# Supplementary material for: Metappuccino: large language model-driven reconstruction of sequence read archive metadata for cancer research
Source: Bioinformatics. 2026 Apr 29;42(5):btag166. doi: 10.1093/bioinformatics/btag166 (PMC13148957; doi:10.1093/bioinformatics/btag166)
Supplement: btag166_Supplementary_Data [file btag166_supplementary_data.zip › BIOINF-2025-2575.R1-suppl.pdf]

Supplementary Material

1. Initial SRA composition of fields extracted by Metappuccino

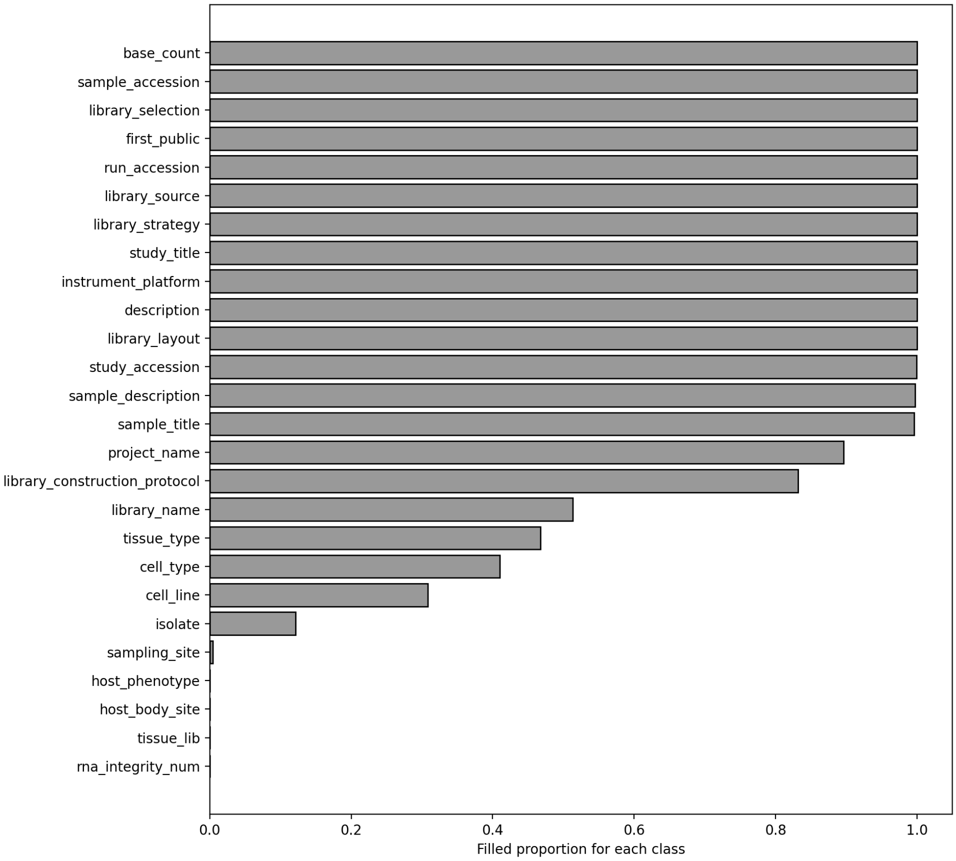

Figure 1 Proportion of missing information in SRA fields used by Metappuccino as input. *computed over 825 476 SRA samples filtered by:* `taxon = tax_eq(9606)`, `strat = library_strategy="rna-seq"`, `dates = first_public` $\in$ `[2012-01-01, 2024-12-31]`, `plat = instrument_platform="illumina"`, `counts = read_count` $\geq$ `10 000 000`.

2. MetappuccinoLLModel: In-Depth Methods

Training Idea

`Mistral-7B-Instruct-v0.3` is the backbone model used. One *LoRA* adapter (?) is trained per class and plugged onto this backbone when needed: the class key in the prompt (e.g. `"organ"` or `"library_selection"`) deterministically selects the corresponding adapter, which is then swapped by enabling its LoRA weights on the frozen backbone (adapters are cached and activated/deactivated without reloading the base model). The prompt presented in Table **??** is reused. This keeps deployment fast, modular, and memory-efficient.

Training procedure and final adapters selection

At each *step*, the model receives the prompt plus the beginning of the JSON answer (e.g. `{"organ":"`) and is trained to predict the next token(s), i.e. the value. Reference tokens (e.g. `lung`) serve only as supervision targets (Supplementary Figure 2.1-2.). For the received prompts at each step, the model attempts to predict the answer using two components: the LM head and the classification head. For open-vocabulary classes, only the LM head, native to the Mistral's architecture, is used. It simply predicts the next tokens until a complete answer is produced. This answer is then compared to the ground truth, and a cross-entropy loss is computed on the answer. The loss is a numeric score that tells how far the model's outputs are from the correct answers. Lower loss means the model's predictions match the targets better. And a cross-entropy loss is the negative log-likelihood of the true label under the model's predicted probabilities, so it is small when the model gives high probability to the correct class and large when it does not. Then, for discrete classes a classification head was added only for the training

phase to help the LM Head. It makes it possible to compute the probabilities for each value that the class can output and therefore has a certain weight in the loss depending on the stage of training, to push it to really choose among the possible classes (Supplementary Figure 2.3-4.). Finally, depending on the outputs, the weights of the LoRA matrices are changed, and it is these weights that form the adapters the user downloads to run MetappuccinoLLModel, in addition to the original Mistral weights (Supplementary Figure 2.5.).
Validation reuses the same prompt with greedy decoding (the model selects the token with the highest probability *argmax*) and its performances are computed by soft or normal accuracy as described in the section *Performance Evaluation*. Early stopping and checkpoint selection saves the model that achieved the highest accuracy on the validation sets for each class. Hyperparameters and settings are documented in the Hugging Face configs of the released adapters (`https://huggingface.co/chumphati/MetappuccinoLLModel`).

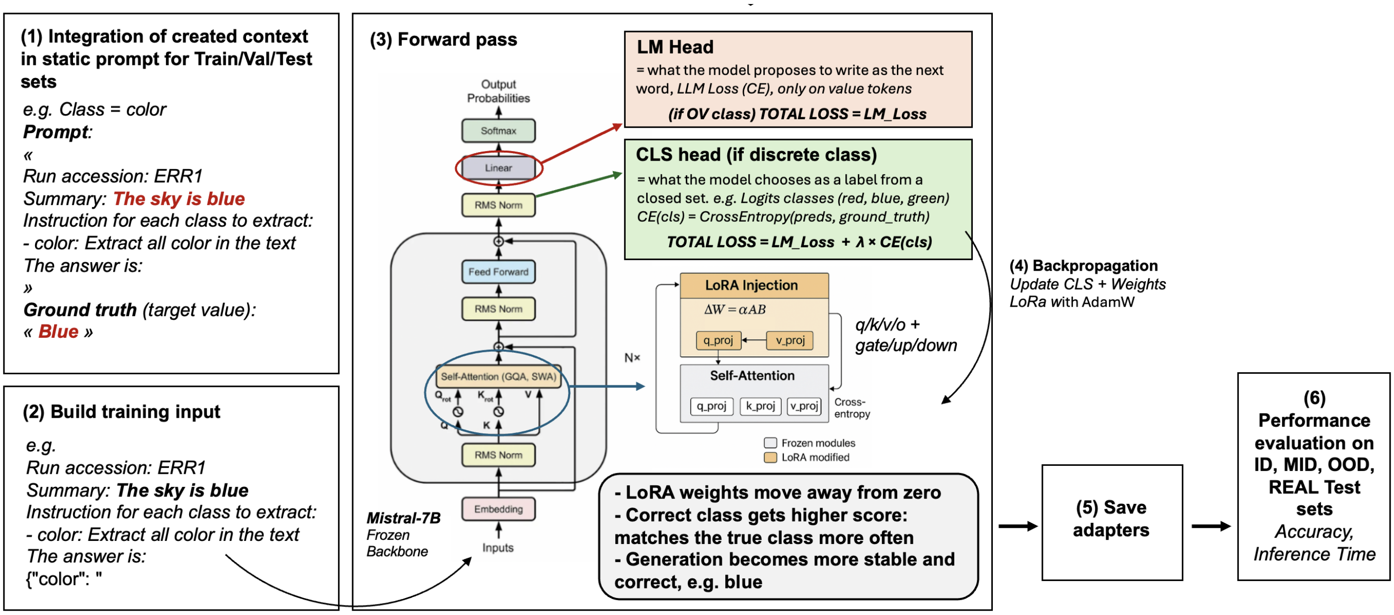

Figure 2 MetappuccinoLLModel Training Workflow. (1–2) Inject the data into the prompt and prepare the model input. (3) Run a forward pass with the untuned Mistral-7B (frozen weights): an LM head generates the next token, and an added classification head for discrete classes computes probabilities over a finite set of values. Ground truth is used to determine whether a prediction is good or not. (4) Backpropagation updates only the LoRA parameters, leaving the base model weights unchanged. (5) Save the updated LoRA matrices during training. (6) During inference, the original model is used, and the saved adapters are activated whenever their associated class must be predicted, to assist the base model.

Data Generation

A key part of training the model and objectively evaluating its performance is to obtain a clean, unbiased dataset.

Extracting diverse data for model training

Since MetappuccinoLLModel's task involves extracting information that is rarely well-structured, obtaining sufficiently large, clean, and varied labeled datasets is difficult. Sentences with explicitly annotated fields tend to follow a repetitive and simplistic pattern (e.g. "class name: value"), which leads models to memorize these patterns rather than learning how to infer information from context. To overcome this limitation, synthetic data was generated. Real SRA metadata were used as the foundation, and the original values of the classes (unknown because they were not annotated) were removed while new ones were inserted. To do so, metadata text blocks were stripped of any words or expressions that could correspond to the 15 classes, resulting in contextual templates resembling authentic metadata but devoid of explicit answers. Each removed element was replaced by a placeholder indicating the class name (e.g. "CELL_LINE"), allowing controlled substitution of class-specific values (Supplementary Figure 3). This approach ensured balanced representation across all classes and produced synthetic data that accurately mirrored the linguistic structure of the real SRA metadata.

Template generation

Two complementary strategies were applied to construct these templates: (1) a manually curated approach (around one hundred templates, divided in 3 sets to not have the same templates for the following train, validation and tests subdivisions) available in the data section of the GitHub repository) (Supplementary Figure 3.1.) and (2) an automated approach combining rule-based methods with large language model reformulation (using Qwen to avoid bias toward Mistral's phrasing) (Supplementary Figure 3.2.). This automated procedure enables large-scale generation and reduces structural redundancy across datasets that mechanically happens with limited, manually curated data. However, this method occasionally fails to remove all ambiguous mentions (for example, implicitly suggesting bulk sequencing could be hard to remove

consistently).
To generate those templates, (1) 800 000 human RNA-seq SRA metadata run accessions were collected and then deduplicated to retain one run per study (38236 samples). Then, (2) the remaining samples were downloaded and summarized with Metappuccino preprocessing steps. (3) Those summaries were then embedded using Mistral, so each row is a normalized vector for one run. (4) For training, we need a representative sampling of the whole pool, to build train and validation sets. We chose to generate clusters to approximate modes/themes in the population. To do that, we did a PCA (n components is 64) to denoise and make distances more meaningful. Then clusters were formed using MiniBatchKMeans on the PCA projection, using Euclidean distance. (5) From these clusters, 3000 training samples were chosen with cluster-proportional quotas method, taking mostly near-centroid points (the core) plus a smaller fraction of peripheral points to cover diversity (Supplementary Figure 3). 500 validation and 1000 tests samples were drawn from the remainder (after removing train points), matching the cluster distribution of the remaining pool, but biased slightly toward the periphery compared to train (Supplementary Figure 3). This allows to capture structurally a majority of the diversity of the known human RNA-seq metadata contexts. These 3500 samples are then automatically processed as explained previously and transformed into templates. Combined with about one hundred manually curated templates, these 3600 templates formed a base to generate good training datasets for each class.

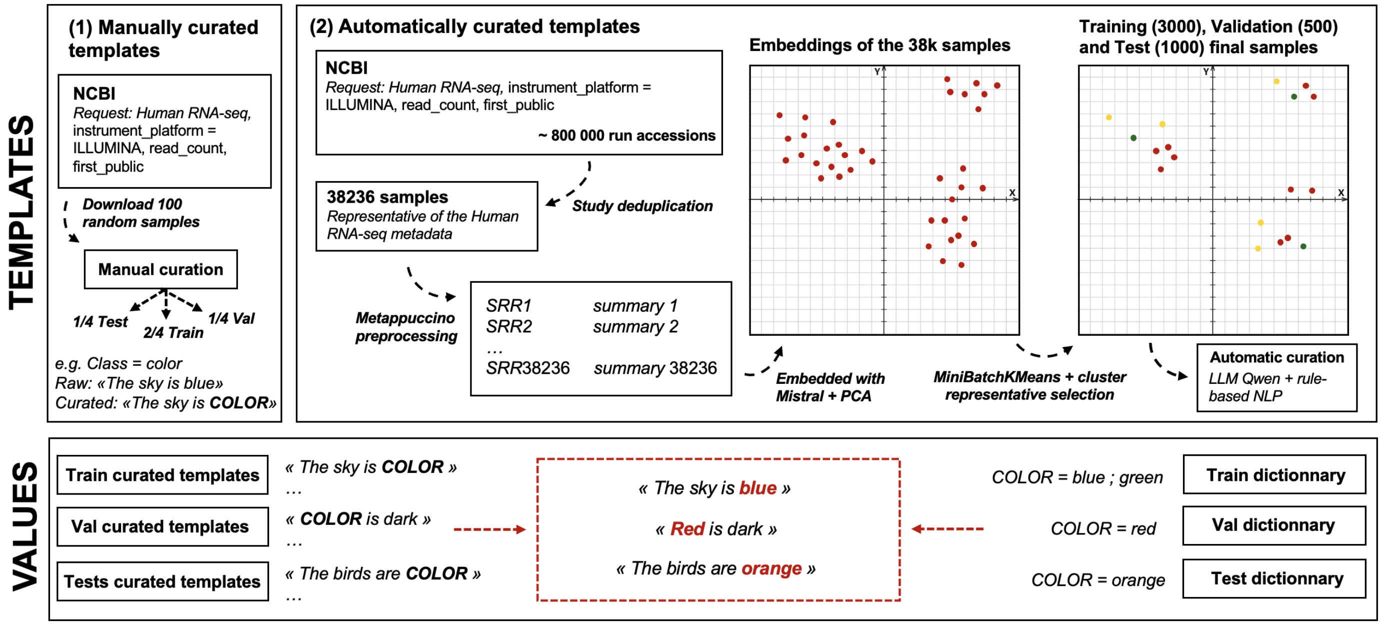

Figure 3 Methods to create the training and evaluation datasets. Synthetic data is created from real SRA data that is cleaned either manually (1) or through automated processes (2). Automated data cleaning can introduce errors but allows for much greater diversity in the data and, above all, data that is representative of our population.

Value integration and dataset balancing

To fill the templates, biologically coherent dictionaries (available on Github) were manually constructed to ensure consistency among classes (e.g. associating "lung" with a liver disease). Values, including unknown were integrated to achieve balanced representations across all classes (Supplementary Figure 9, 3). Discrete classes shared identical value sets across all splits, while open-vocabulary classes contained at least twenty distinct values per class and per dataset, each associated with synonyms (more or less clear) to introduce linguistic diversity. When no placeholder existed for a class, corresponding information was inserted randomly into the context. The same value could appear multiple times depending on the number of detected placeholders. For open-vocabulary classes, no value overlap existed between training, validation, and test sets, ensuring that models learned to infer meaning rather than memorize specific values. For "unknown" instances, we intentionally insert no evidence for that attribute in the synthetic text, so correct performance requires detecting that the information is missing and abstaining accordingly.

Per-class Train-Set & Validation-Set

Different metadata classes responded differently to input sources. Some benefited more from automatically generated templates, whereas others performed better with manually curated data. Consequently, train and validation compositions varied by class (Supplementary Figure 7, 4.1.). `library_selection`, `biopsy_site`, `organ`, `disease`, and `sex` were trained on 2990 synthetic examples built from automatically generated templates, with 499 validation examples constructed in the same way but using distinct values. The class `biopsy_type` required additional rebalancing (blood, primary, metastasis) and therefore used 1000 training and 300 validation examples with different values but was still

built with the automatic templates. In contrast, `sequencing_source`, `cell_line`, `cell_type`, `age`, `treatment`, `treatment_time`, `response`, `is_cancer`, and `ethnicity` achieved better results when trained on mixed datasets combining synthetic and real SRA contexts. Depending on data availability, 20–50% of their training data consisted of authentic SRA metadata with reliable annotations. However, 100% real data were avoided to prevent overfitting on trivially annotated patterns (e.g. "class name: value"). The remaining part of training sets and the validation sets for these classes were built from manually curated templates (duplicated to get at least 1000 training data) (Supplementary Figure 4.1.). Supplementary Figure 9 reports, for each class, the number of values used and their distribution across the train, validation, and test sets.

Per-class synthetic Test-Sets

To provide robust evaluation, 3 test splits were created for each class: in-distribution (ID), out-of-distribution (OOD), and middle-distribution (MID) (Supplementary Figure 4.2.). ID samples had contextual embeddings close to their associated training sets and thus were expected to yield the best performance. OOD samples were chosen for maximal distance in embedding space and represented structurally distinct contexts, where performance was expected to decline. MID samples occupied intermediate positions, providing insight into model behavior across gradual distribution shifts. Each split contained 400, 400, and 1,200 samples respectively (Supplementary Figure 7, 4.2.). For every class, two raw test sets were generated by filling the test automatic (1000) and manual (1000) templates with new equilibrated classes values (2000 total) Figure 4.2.). 30% of class values were added in a clear way (the answer pasted directly into the text), and 70% were added in a masked way by mixing in synonyms or noise so the information is harder to find (which roughly represents the distribution of whether the SRA labels are filled in or not). Those 2000 synthetic samples where then separated into the 3 test subsets (ID, MID, OOD) according to their embedding similarity to the training data using Mistral embeddings by separability analysis (AUC-based classification). The AUC is computed from the embeddings by (1) calculating the training centroid (the L2-normalized mean vector of the L2-normalized training embeddings), (2) measuring the cosine similarity between this centroid and the embeddings of each dataset to be compared. The higher the cosine score, the closer the dataset to compare is to the training distribution, and conversely. This is why AUC(test ID) ¿ AUC(test MID) ¿ AUC(test OOD) is expected, which is indeed observed (Supplementary Figure 7). The coexistence of manual and automatic templates naturally facilitated structural diversity: when a model was trained on automatically generated templates, manually constructed ones served as ideal OOD tests, and conversely.

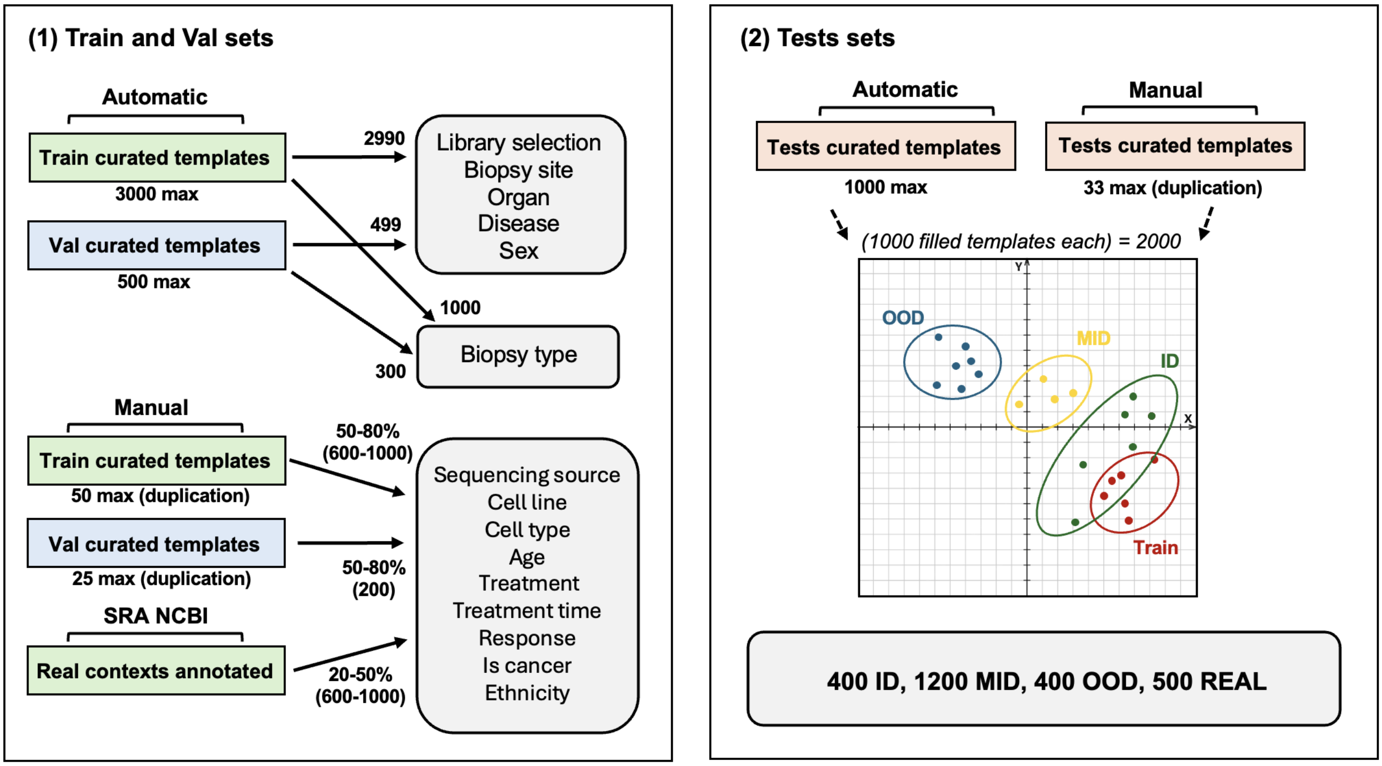

Figure 4 Dataset split methods. (1) Each Metappuccino class is trained on distinct datasets composed of templates generated by different methods, selected based on how well they capture the target signal. This figure reports, for each class, the distribution of template types across the training and validation splits. Numbers on the arrows indicate the count or proportion of each template type, and the number beneath each template block gives the total number of templates of that type produced by the methods in Figure 3. (2) Test-set split. From each template type, 1000 samples were generated, so 2000 test samples in total. For each class, each sample's distance was computed to its own training set: the 400 nearest are labeled ID, the 1200 in the middle are MID, and the 400 farthest are OOD.

Real Test-Set

In addition to the previously described synthetic test datasets, a real dataset was created. This set comprised 400 randomly selected SRA contexts that were disjoint from training at the run-accession level, plus 100 additional contexts from a melanoma case study that were also randomly selected. As shown in Figure 5, this set spans a wide range of submission dates and is largely study-diverse (most runs come from distinct studies, used here as a proxy for submitter style), with only a small tail of multi-run studies that mainly arises from the melanoma subset. Because real SRA records do not provide complete reference labels for the 15 classes, accuracy cannot be computed. Ground truth was therefore established by manual annotation of all 500 samples using only the inputs available to Metappuccino, and no additionnal Cellosaurus dictionnaries (Figure **??**). For the 100 melanoma samples, a second reference was produced by complementing the previous labels with Cellosaurus-based information consistent with Metappuccino's pre and postprocessing logic, which enabled performance evaluation of both the LLM inference and the end-to-end system (*Study case with Metappuccino : 100 melanoma samples* section).

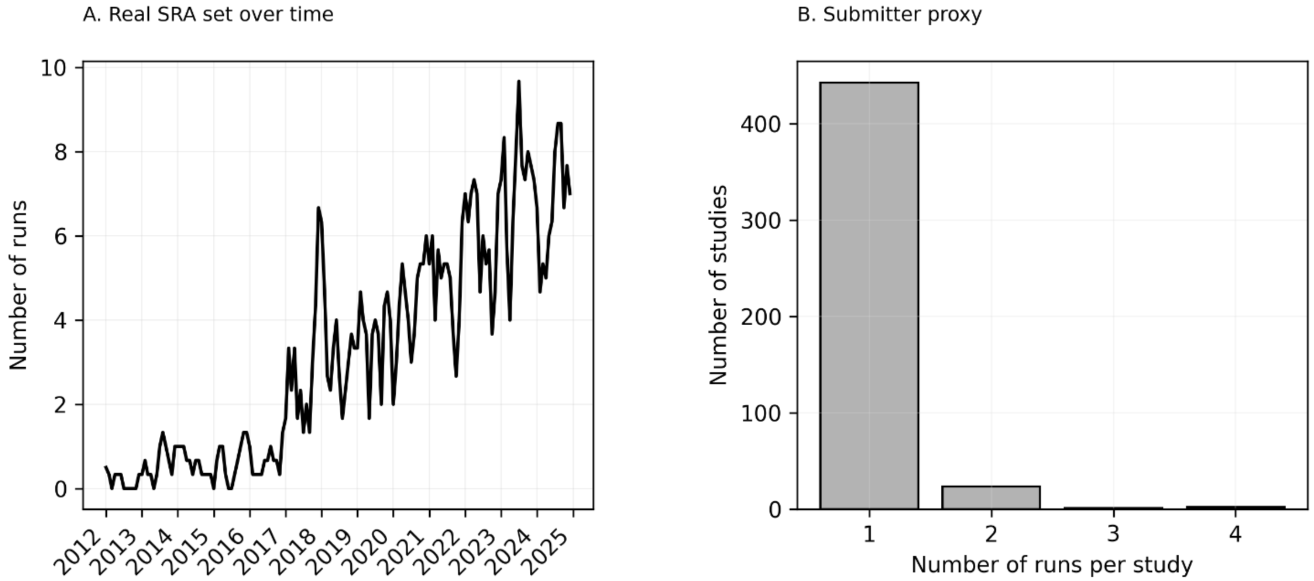

Figure 5 Overview of the composition of the 500-real SRA test set. A. Temporal distribution of run publication dates. B. Study-level clustering in the same set, shown as the number of runs originating from the same study; most studies contribute a single run, with a small tail of multi-run studies.

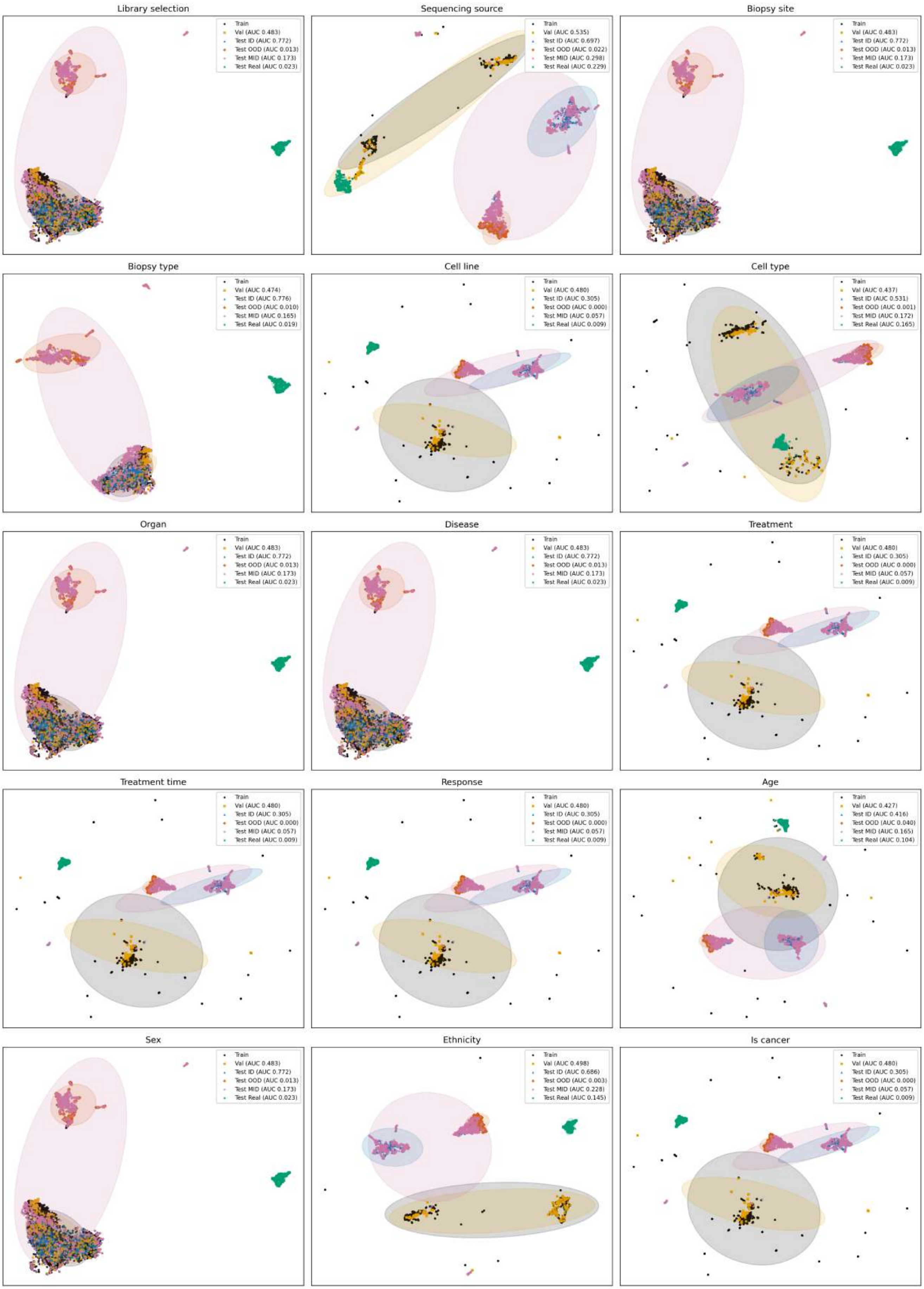

Figure 7. Embedding distributions across all metadata classes. Two-dimensional UMAP projections of the L2-normalized Mistral embeddings for training (black), validation (orange), and test splits (ID = blue, MID = pink, OOD = red) plus real SRA contexts (green). Ellipses represent confidence regions. The AUC values indicate cosine-based separability between the training centroid and each split: higher AUC reflects stronger similarity. MID and ID sets show moderate overlap, while OOD and real contexts form distinct clusters, confirming controlled distribution shifts and minimal overlap across splits.

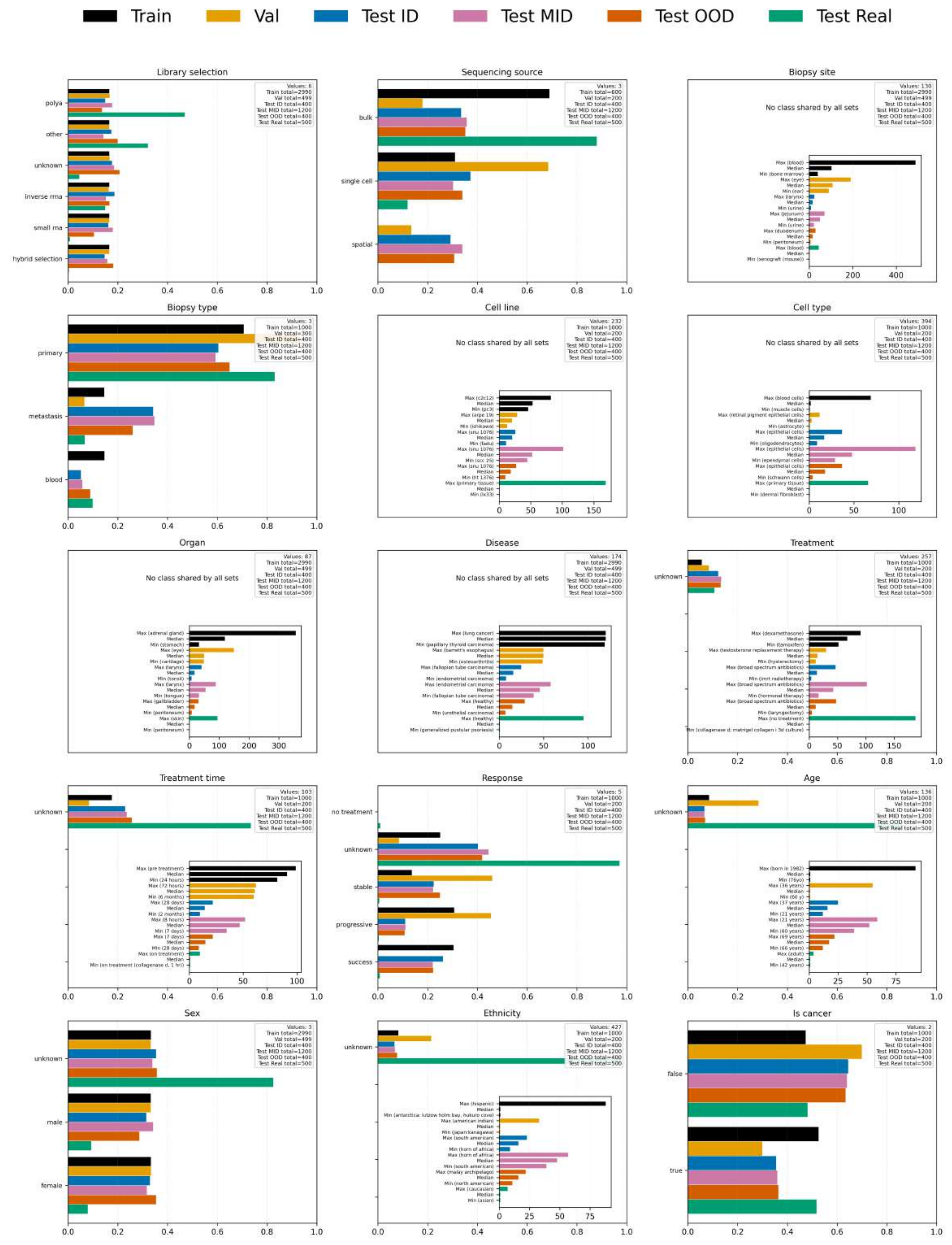

Figure 9. Class distributions across all metadata classes. Distribution of class values per split (Train, Validation, Test ID, Test MID, Test OOD, and Test Real) for each metadata class. Synthetic datasets were filled using biologically coherent dictionaries to ensure internal consistency and were balanced as evenly as possible across splits through controlled template generation. Open-vocabulary classes include around 20 distinct synthetic values per dataset. Classes displaying a larger number of values correspond to those partially trained on real SRA metadata, where authentic annotations were integrated into the training and validation sets. Thoses values are listed in a JSON file available in the data section of the Github project. When perfect balance was not achievable, training algorithms were adjusted to handle the imbalance classes.

Data Leakage

Contexts and values were disjoint across train, validation, and test splits, which reduced direct overlap. To further verify the absence of leakage due to overly similar contexts, two embedding-based diagnostics were computed per class and per split (Supplementary Figure 10): (1) A distributional shift was measured with the squared Maximum Mean Discrepancy (MMD). This measures how far apart the overall shape of a dataset's points is from the training points: smaller values mean the two distributions are more similar. The comparison uses distances based on cosine similarity, and a Gaussian smoothing factor whose width is automatically chosen using the median of all pairwise distances. (2) The near-duplicates: for each dataset item, the most similar training item is found using cosine similarity. If this maximum similarity is very high (more than 0.95), the test item is counted as a near-duplicate (ND). ID splits show low MMD as expected (close from training by nature), MID are intermediate, and OOD and real-world tests exhibit larger MMD, consistent with intentional distributional shift and embedding distributions (Supplementary Figure 7). Depending on the class, MID and ID can show very similar MMD, and MID can even be lower. This is expected because MID usually has almost 3 times more samples, which reduces the variance of the empirical MMD estimate, and in some classes MID is genuinely closer to the train distribution. Near-duplicate rates are essentially zero in test splits, with only small residuals on a few validation sets (e.g. age), which aligns with constrained phrasing in some classes. Those observations reflect heterogeneous training distributions, especially when real SRA contexts are part of the training sets, which expands the coverage of the training embedding space and can raise baseline MMD without implying leakage. Overall, the diagnostics support minimal data leakage and a clean separation between in-distribution and shifted test conditions.

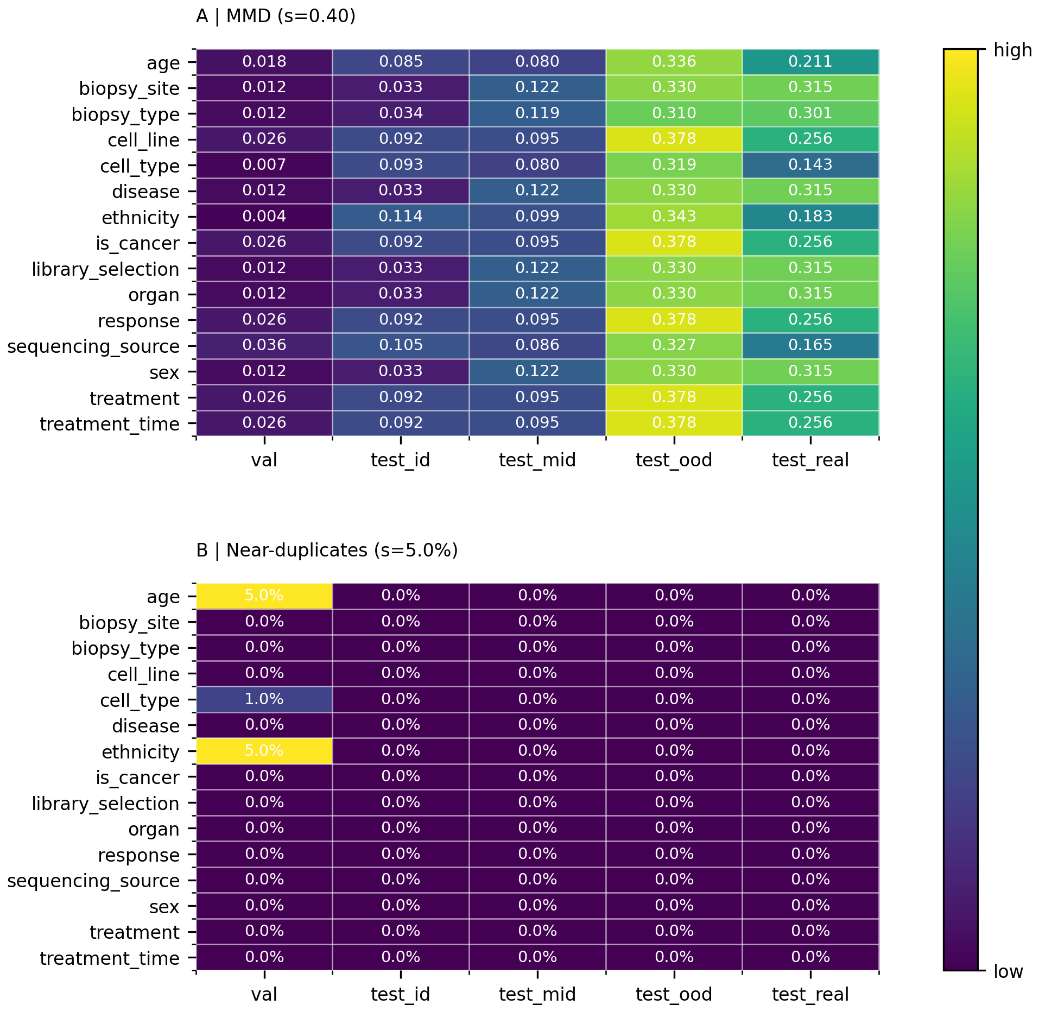

Figure 10 Data leakage evaluation between train, validation, and test sets. *MMD (A).* Small values mean that the set has similar distributions to the train. *Near-duplicates (B).* The table reports the percentage ND, so values near zero indicate that copy-like items are absent.

General Performance Benchmarking

Performance evaluation

(1) Soft accuracy for context-tolerant classes Given a set of N samples, $p_i \in \mathbb{R}^d$ is the embedding of the model's prediction and $r_i \in \mathbb{R}^d$ the embedding of the reference metadata for sample i. These embeddings are obtained by encoding each text string with a sentence-transformer model (*pritamdeka/BioBERT-mnli-snli-scinli-scitail-mednli-stsb*), which maps semantically medical similar terms to nearby points in the d-dimensional space. The cosine similarity between p_i and r_i is $\text{sim}(p_i, r_i) = \frac{p_i \cdot r_i}{\|p_i\| \, \|r_i\|}$, where the numerator is the dot product and the denominator the product of Euclidean norms. A higher value (up to 1) indicates stronger semantic alignment. If $\text{sim}(p_i, r_i) \geq \tau$, the prediction is counted as correct. Thresholds τ were set empirically, searching where the right/wrong switch occured in the comparison reference/predictions (e.g. ref='True', prediction='False', score=0.3, $\tau = 0.2$, answer='matching'. Here, the answer 'matching' is wrong, higher thresholds are needed to get answer='non matching'). Finally we founded that: $\tau_{\text{default}} = 0.40$, $\tau_{\text{disease}} = 0.42$, $\tau_{\text{organ}} = 0.37$, $\tau_{\text{ethnicity}} = 0.30$, $\tau_{\text{treatment}} = 0.33$, $\tau_{\text{treatment\_time}} = 0.35$. The default applies to all other classes. The semantic accuracy at threshold τ is then

$$\text{Acc}_{\text{sem}}(\tau) = \frac{1}{N} \sum_{i=1}^{N} \mathbf{1}\big[\text{sim}(p_i, r_i) \geq \tau\big].$$

(2) Classical accuracy for exact-matches classes For classes requiring strict identity, strings are lowercased and punctuation is removed before comparison, and a prediction is correct only if the normalized strings are identical.

Additional results

From explicit fields to fuzzy cues: variation in MetappuccinoLLModel inference performance

To test whether our synthetic benchmarks reduce to a simple "recover the inserted label" exercise, we designed a controlled evaluation in which only the surface form of the target value changes while the underlying class to predict remains identical. Concretely, we reuse the same synthetic templates as decribed in the previous paragraphs (n=2000) and, for each metadata class, we insert the same target label in two variants: a *clear* variant, where the label is inserted in its explicit, canonical wording, and a *syn* variant, where the very same label is inserted via an alternative surface form (synonym or paraphrase) that expresses the same class with different wording. The complete mapping between synonymized strings and canonical labels is provided in our GitHub repository. We then evaluate, class by class, whether the fine-tuned model predicts the correct canonical label from the resulting template text under these two insertion regimes. Figure 11 shows that replacing clear label insertions by synonymized insertions induces only a moderate decrease in performance for most classes, indicating that the model's score is not primarily driven by trivial recovery of a single canonical string, but also by mapping varied realizations back to the correct label. In contrast, for the classes that are already the most difficult in our main analyses (library selection and treatment), the performance is more sensitive to how the label is expressed: when the target value is inserted in a more indirect or paraphrased form, errors increase and uncertainty rises. This behavior mirrors our manual evaluation on real SRA records, where the same classes concentrate most failures when the available text is incomplete or ambiguous. Overall, this control suggests that the aggregate synthetic score is not dominated by trivially recoverable cases, since comparable label recovery is maintained even when the inserted target values are expressed through non-canonical surface forms.

Handling missing information

Beyond recovering labels that are supported by the input text, a key aspect of metadata completion is knowing when the context does not justify a specific value. We therefore evaluate the model's ability to use *unknown* appropriately, treating it as a dedicated class. In our synthetic setup used for the *From explicit fields to fuzzy cues: variation in MetappuccinoLLModel inference performance* analysis, whenever the gold label for a field is *unknown*, we deliberately leave that slot unlabeled (no category-specific label is inserted, in either the *clear* or *syn* variant), so that the correct behavior is to abstain rather than guess. Figure 12 reports *unknown* precision (how often a predicted *unknown* matches an *unknown* gold label) and *unknown* recall (how often *unknown* gold labels are predicted as *unknown*) on the unknown-balanced subset of this dataset. Across most categories, recall is high, suggesting that the fine-tuned model often refrains from filling in unsupported values: it can recognize many context-unsupported cases. Precision is more heterogeneous, indicating that the model still sometimes defaults to *unknown* even when a value is present in the template, a limitation that matches the error patterns observed in our main analyses. As expected, this sensitivity is strongest for already challenging attributes: when inserted labels are less explicit, the model is more likely to miss them and fall back to *unknown*.

MetappuccinoLLModel confidence on tested inference instances

To characterize model confidence on real SRA inputs, we analyzed the distribution of predictive uncertainty scores produced during inference on the 500-run manually curated test set. For each predicted field, we compute predictive entropy from the model's token-level probabilities (the same quantities used to derive NLL/PPL in our outputs), and summarize these values per metadata category (Figure 13.A.) and across all runs and categories (Figure 13.B.). Within a given model, lower entropy means probability mass is concentrated on a small set of candidate outputs (more decisive predictions), whereas higher entropy reflects more dispersed probabilities (less decisive predictions). Most predictions fall in a low-entropy range, with a smaller tail of higher-entropy cases. Because entropy is computed in each model's own tokenization space,

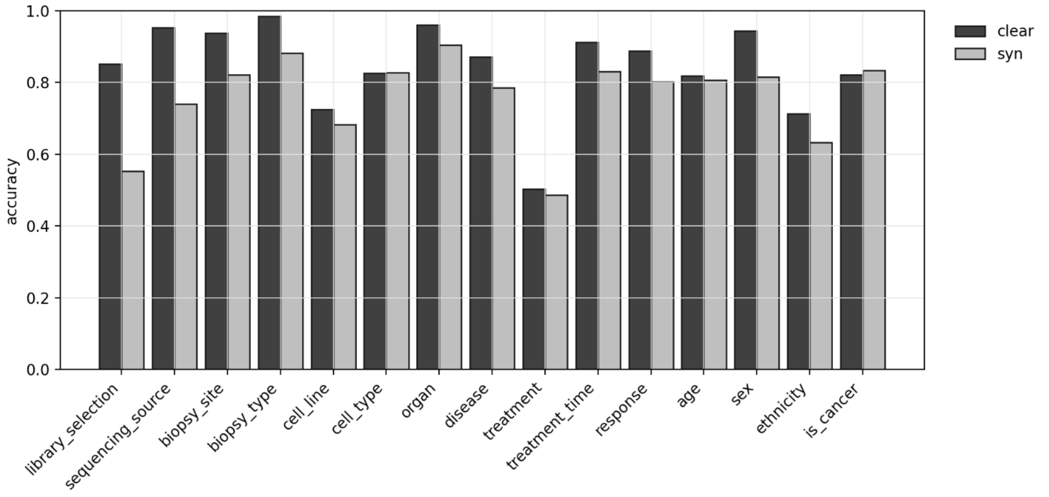

Figure 11 MetappuccinoLLModel inference accuracy for clear vs synonymized inserted metadata labels. For each metadata category, we report the fraction of runs where the model's prediction matches the gold label. "clear" corresponds to evaluation examples where the gold label is inserted into the prompt template in its canonical, explicit form, whereas "syn" corresponds to the same labels inserted using a synonymized or paraphrased surface form (same underlying class, different wording). The list of synonyms used for all categories are available in the section *Data* in Metappuccino's Github.

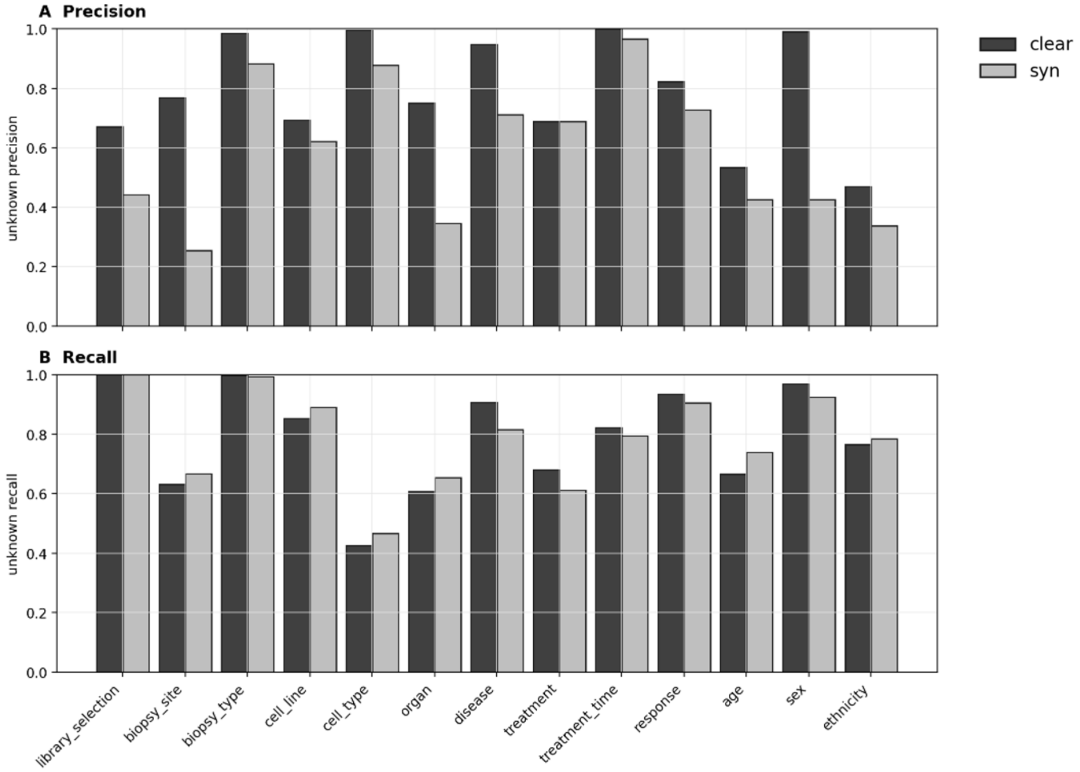

Figure 12 Unknown predictions for clear vs synonymized metadata labels. A. Unknown precision across categories (how often predicted unknown corresponds to an actual unknown gold label). B. Unknown recall across categories (how often unknown gold labels are predicted as unknown).

absolute values are not comparable across models; we therefore use it only for qualitative inspection and within-model ranking. In particular, these results support using entropy as a practical per-instance flag for manual review (high-entropy outputs), while not treating low entropy as a guarantee of correctness, since some fields can be confidently wrong and others correct despite higher uncertainty.

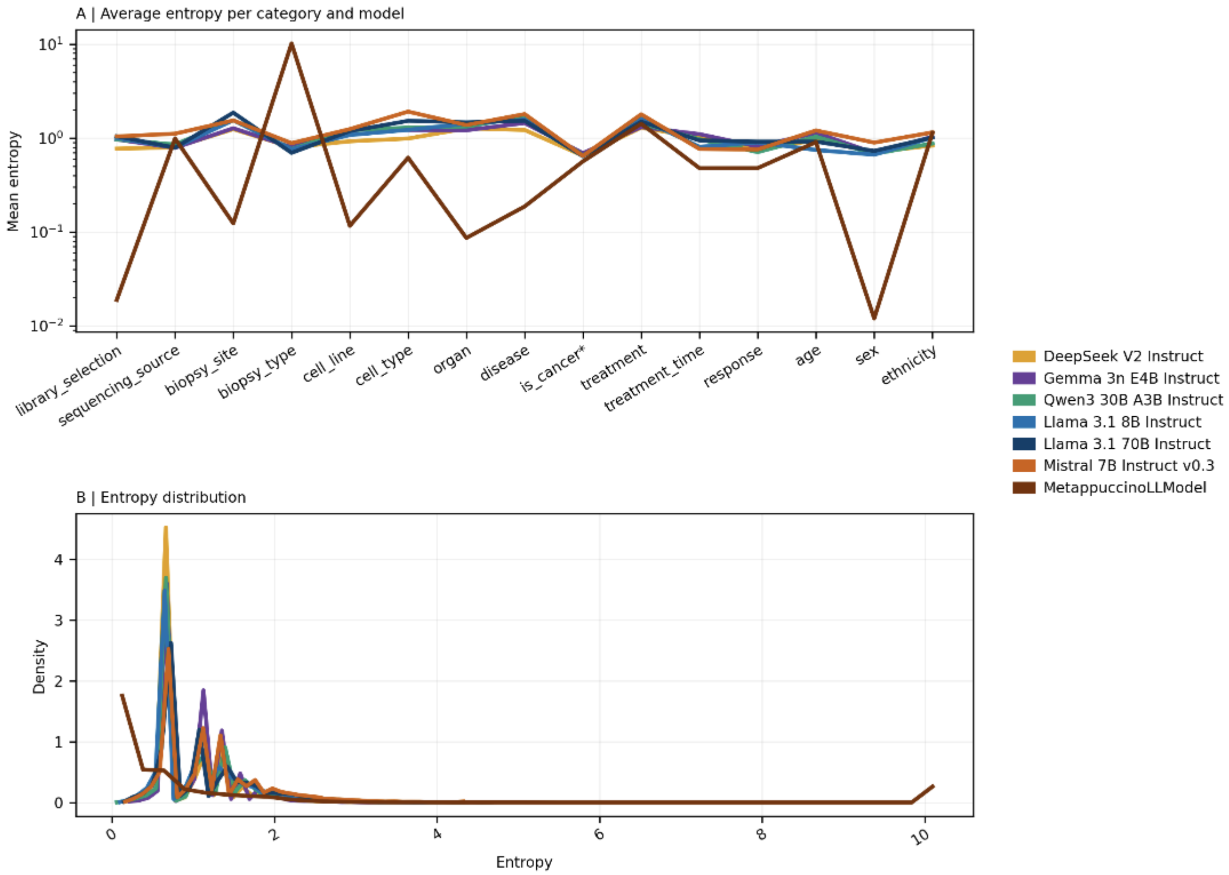

Figure 13 Predictive entropy of LLM metadata inferences across categories and models. A. Mean predictive entropy per metadata category for each evaluated model on the 500-run real SRA test set. Higher entropy indicates greater uncertainty for that category. B. Density of entropy values across all categories and runs, showing how each model distributes its confidence mass over low vs high uncertainty predictions. *Note: Because entropy is computed from each model's native token probabilities (and tokenization differs across models), absolute entropy values are not strictly comparable between models; comparisons are most reliable within a model across categories.*

Disagreements between MetappuccinoLLModel inferences and preprocess-extracted values

On the 500-run manually curated real SRA test set, we compared Metappuccino's deterministic preprocessing outputs (rule-based extraction) to the LLM predictions by evaluating both against the same gold standard and using the same semantic match criterion (cos_sim ≥ 0.40). Figure 14.A. reports per-field accuracy for extraction and for the LLM, restricted to the subset of runs where a deterministic value could actually be extracted; n denotes the size of this subset for each field. This restriction matters because extraction coverage varies widely across categories: for several fields, preprocessing yields few (or no) extracted values on this test set, and categories not shown simply correspond to ¡10 extraction coverage in these 500 runs (Figure 14.A.).

Beyond fields where extraction and the LLM achieve similar accuracy, Figure 14.A. also highlights categories where they behave differently. In several cases, the LLM attains higher accuracy than deterministic extraction on the same extracted subset, suggesting that it can better interpret heterogeneous or loosely stated evidence even when an extracted candidate value exists. Conversely, for a small number of fields, most notably library_selection, deterministic extraction remains more reliable than the LLM when it applies, consistent with the higher mismatch rates observed for these fields in Figure 14.B. Figure 14.B. quantifies how often the LLM disagrees with the deterministic extraction (cos_sim < 0.40) and shows that disagreements are field-dependent rather than uniform; the inset boxplots further indicate that LLM–extraction agreements tend to coincide with more confident LLM outputs than disagreements (Figure 14.B.).

These results directly informed Metappuccino's per-field resolution strategy. When extraction is judged reliable for a given category, we retain it; when extraction is uncertain or inconsistent with the aggregated context, we pass the extracted candidate to the LLM as additional input and let the LLM arbitrate. In practice, because Figure 14.A. shows higher LLM accuracy for most fields, the LLM is used as the default resolver, while extraction is preferred for library_selection and treatment, where it is empirically more accurate on this real test set (Figure **??**, 14.A.).

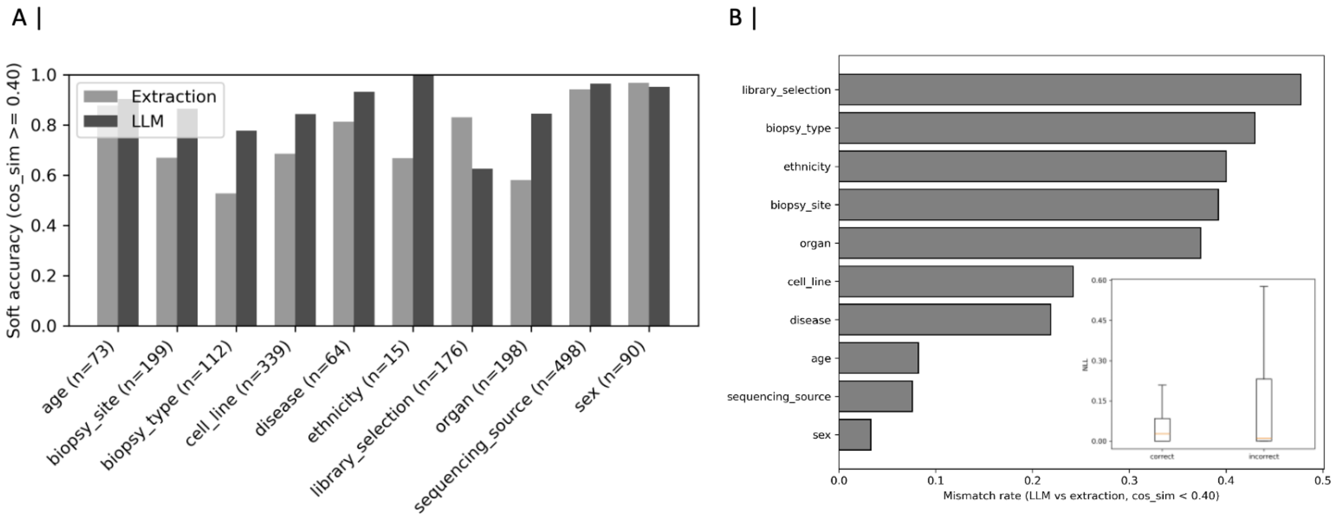

Figure 14 MetappuccinoLLModel vs extraction. A. Field-wise semantic agreement between the LLM output and the rule-based extraction; sample sizes per field are shown in parentheses. B. Mismatch rate by field, highlighting the metadata categories where the LLM most frequently diverges from deterministic extraction. The boxplot shows the distribution of the LLM's uncertainty scores (NLL) for cases where it agrees with the extraction (correct) versus where it disagrees (incorrect).

2. Metappuccino

Extracted fields from the NCBI API during pipeline download

The downloaded BioSample XML file contains sample-level record with attributes under the corresponding tag, with keys taken from `attribute_name` or `harmonized_name`) to capture details often missing at run level. In parallel, a call to the SRA API provides the following fields: `study_accession`, `first_public`, `study_title`, `project_name`, `run_accession`, `sample_accession`, `sample_title`, `sample_description`, `library_name`, `library_selection`, `library_source`, `library_strategy`, `library_construction_protocol`, `library_layout`, `rna_integrity_num`, `instrument_platform`, `rt_prep_protocol`, `cell_line`, `cell_type`, `tissue_lib`, `tissue_type`, `host_phenotype`, `isolate`, `age`, `host_body_site`, `sampling_site`, `base_count`, `description`.

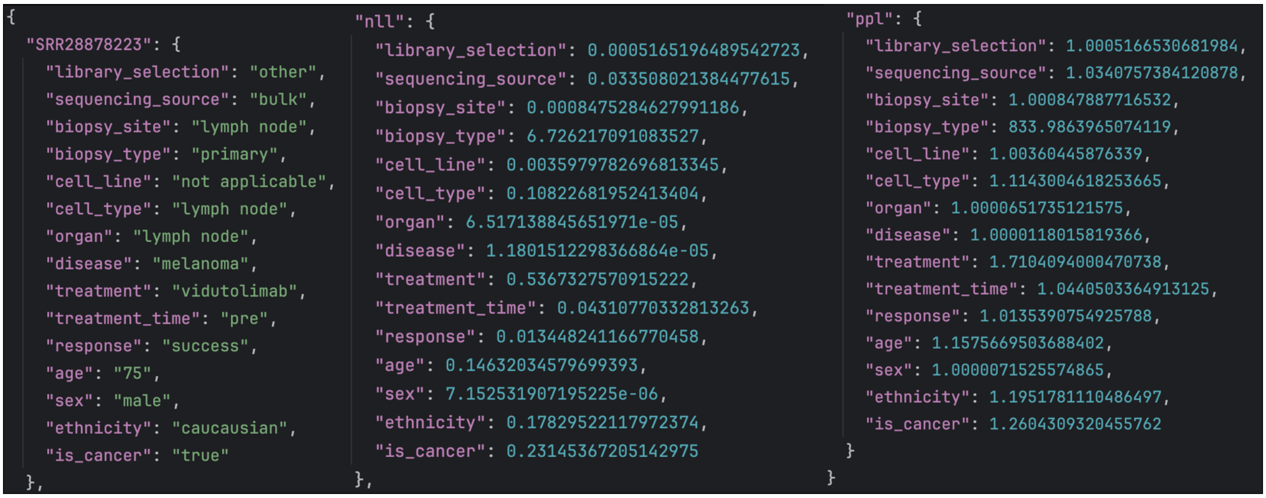
{
  "SRR28878223": {
    "library_selection": "other",
    "sequencing_source": "bulk",
    "biopsy_site": "lymph node",
    "biopsy_type": "primary",
    "cell_line": "not applicable",
    "cell_type": "lymph node",
    "organ": "lymph node",
    "disease": "melanoma",
    "treatment": "vidutolimab",
    "treatment_time": "pre",
    "response": "success",
    "age": "75",
    "sex": "male",
    "ethnicity": "caucausian",
    "is_cancer": "true"
  },
  "nll": {
    "library_selection": 0.0005165196489542723,
    "sequencing_source": 0.033508021384477615,
    "biopsy_site": 0.0008475284627991186,
    "biopsy_type": 6.726217091083527,
    "cell_line": 0.0035979782696813345,
    "cell_type": 0.10822681952413404,
    "organ": 6.517138845651971e-05,
    "disease": 1.1801512298366864e-05,
    "treatment": 0.5367327570915222,
    "treatment_time": 0.04310770332813263,
    "response": 0.013448241166770458,
    "age": 0.14632034579699393,
    "sex": 7.152531907195225e-06,
    "ethnicity": 0.17829522117972374,
    "is_cancer": 0.23145367205142975
  },
  "ppl": {
    "library_selection": 1.0005166530681984,
    "sequencing_source": 1.0340757384120878,
    "biopsy_site": 1.000847887716532,
    "biopsy_type": 833.9863965074119,
    "cell_line": 1.00360445876339,
    "cell_type": 1.1143004618253665,
    "organ": 1.0000651735121575,
    "disease": 1.000011801581936,
    "treatment": 1.7104094000470738,
    "treatment_time": 1.0440503364913125,
    "response": 1.0135390754925788,
    "age": 1.1575669503688402,
    "sex": 1.0000071525574865,
    "ethnicity": 1.1951781110486497,
    "is_cancer": 1.2604309320455762
  }
}

Figure 15 Example of raw JSON output after LLM inference: SRR28878223. The file has three top-level keys: the run name, the negative log-likelihood (nll) and the perplexity (ppl). It reports results for a single sample at inference time; these values are later merged by Metappuccino with normalization and pre/postprocessing into the final output. The nnl and ppl quantify the uncertainty of a model when predicting the next token in a sequence. For each class, the model outputs a short sequence of T tokens, written $y_{1:T}$. At each step t, it assigns a probability p_t to the token it emits. The *token-level uncertainty* is computed by $s_t = -\log p_t$. Then, the average of s_t is taken: $\text{nll} = \frac{1}{T}\sum_{t=1}^{T} s_t$. Perplexity is defined as $\text{ppl} = e^{\text{nll}}$. s_t and kept to facilitate interpretability. Lower nll and ppl indicates higher confidence.
